# ALA_PDT Promotes Ferroptosis-Like Death of *Mycobacterium abscessus* and Antibiotic Sterilization via Oxidative Stress

**DOI:** 10.3390/antiox11030546

**Published:** 2022-03-14

**Authors:** Xiaoyu Wang, Meiyin Wan, Lei Zhang, Yongdong Dai, Yang Hai, Chenda Yue, Junqi Xu, Yadan Ding, Mei Wang, Jianping Xie, Xia Lei, Julia-Li Zhong

**Affiliations:** 1 Key Laboratory of Biorheological Science and Technology, Ministry of Education, College of Bioengineering, Chongqing University, Chongqing 400044, China; 20181901038@cqu.edu.cn (X.W.); 201819021011@cqu.edu.cn (M.W.); 202019021052@cqu.edu.cn (Y.D.); wangmei@zmu.edu.cn (M.W.); 2 Department of Dermatology, Daping Hospital, The Army Medical University, Chongqing 400044, China; yuecd@hotmail.com; 3 Institute of Modern Biopharmaceuticals, State Key Laboratory Breeding Base of Eco-Environment and Bio-Resource of the Three Gorges Area, Key Laboratory of Ministry of Education Eco-Environment of the Three Gorges Reservoir Region, College of Life and Sciences, Southwest University, Chongqing 400700, China; zl19980601@email.swu.edu.cn (L.Z.); a1347889857@email.swu.edu.cn (Y.D.); hy357159@email.swu.edu.cn (Y.H.); jj20200901@swu.edu.cn (J.X.)

**Keywords:** ALA_PDT, *Mycobacterium abscessus*, ferroptosis, heme oxygenase MAB_4773, antibiotic resistance

## Abstract

*Mycobacterium abscessus* is one of the common clinical non-tuberculous mycobacteria (NTM) that can cause severe skin infection. 5-Aminolevulinic acid photodynamic therapy (ALA_PDT) is an emerging effective antimicrobial treatment. To explore whether ALA_PDT can be used to treat *M. abscessus* infections, we conducted a series of experiments in vitro. We found that ALA_PDT can kill *M. abscesses*. Mechanistically, we found that ALA_PDT promoted ferroptosis-like death of *M. abscesses*, and the ROS scavenger N-Acetyl-L-cysteine (NAC) and ferroptosis inhibitor Ferrostatin-1 (Fer-1) can mitigate the ALA_PDT-mediated sterilization. Furthermore, ALA_PDT significantly up-regulated the transcription of heme oxygenase MAB_4773, increased the intracellular Fe^2+^ concentration and altered the transcription of *M. abscessus* iron metabolism genes. ALA_PDT disrupted the integrity of the cell membrane and enhanced the permeability of the cell membrane, as evidenced by the boosted sterilization effect of antibiotics. In summary, ALA_PDT can kill *M. abscesses* via promoting the ferroptosis-like death and antibiotic sterilization through oxidative stress by changing iron metabolism. The study provided new mechanistic insights into the clinical efficacy of ALA_PDT against *M. abscessus*.

## 1. Introduction

Mycobacterial infections can be divided into three main categories: (1) infections caused by *Mycobacterium tuberculosis* or *M. bovis*, (2) infections caused by *M. leprae*, and (3) non-tuberculous mycobacteria (NTM) infection [1]. NTMs are diverse and ubiquitous in the environment, but only very few can cause serious infections, such as *M. abscesses* [2]. As an emerging pathogen worldwide, *M. abscesses* is a rapidly growing NTM [3]. *M. abscesses* is resistant to most anti-tuberculosis drugs, such as macrolides, aminoglycosides, rifampicin, tetracycline and β-lactam antibiotics [1]. Multi-drug resistant *M. abscessus* was developed via mechanisms similar to other NTMs [4], and greatly limited the choice of antibiotics.

New measures against multi-drug resistant *M. abscesses* are intensively pursued, such as phage therapy [5,6], natural or synthetic antimicrobial peptide [7] and photodynamic therapy (PDT) [8]. PDT is an emerging treatment modality for diseases with the combination of photosensitive drugs and corresponding wavelengths of light. PDT can sterilize many microbes in vitro via the combination of photosensitizer and light [9]. PDT was firstly used to treat skin tuberculosis in the late 19th century by Niels Ryberg Finsen, who received the Nobel Prize for phototherapy development in 1903 [10]. Subsequently, many studies on the effect of phototherapy, photochemotherapy and PDT against bacteria were published [11]. However, the discovery and application of antibiotics dwarfed the photodynamic antibacterials [12]. PDT was revitalized largely due to the emergence of drug-resistant bacteria. 5-Aminolevulinic acid (ALA) is a second-generation photosensitizer. It is a key intermediate in the biosynthesis of protoporphyrin IX (PpIX) and heme. Excessive exogenous ALA supply resulted in PpIX accumulation, which can act as a bona fide photosensitizer under irradiation [13]. ALA_PDT has been widely used in the treatment of clinical acne, actinic keratosis, various skin diseases and cancer [14]. PDT was recognized as a promising alternative treatment for drug-resistant tuberculosis or NTMs infection [15], as evidenced by the fact that PDT can effectively inactivate *M. fortuitum* [16], *M. bovis* BCG [17], *M. marinum*, *M. smegmatis* [18] and *M. tuberculosis*. PDT can alleviate granulomas induced by *M. bovis* BCG in vivo [19]. We have previously reported that ALA_PDT can treat mycobacterial skin infection [20].

Although the exact mechanism of PDT remains unclear, the reactive oxygen species (ROS) produced by PDT might underlie its bacteriocidal effect [21]. Two oxidation mechanisms can be attributable to the production of free radicals and singlet oxygen by photodynamics [22]. These active substances can inactivate microorganisms by damaging various cellular components, possibly through the photooxidation of nucleic acids [23], proteins [24,25] and membrane lipids [26,27]. PDT can elicit single-stranded and double-stranded DNA damage in gram-positive or gram-negative bacteria, and abrogate the plasmid supercoil [28]. The DNA damage effect of PDT against bacteria might be minor to the death of microbes largely due to the presence of proficient DNA repair systems [29].

PDT can cause non-enzymatic lipid peroxidation via a distinct ferroptosis-like cell death [30]. Ferroptosis is characterized by lipid peroxidation of cell membranes under the action of iron or lipid oxidase, depleting glutathione, shrinking mitochondria and elevating membrane density [31]. Ferroptosis on eukaryotes is precisely regulated, which directly or indirectly targets iron metabolism and lipid peroxidation [32]. Ferrous and polysulfide can synergistically induce ferroptosis-like death in bacteria [33]. ROS, pivotal for PDT efficacy, can induce cell ferroptosis. However, the relationship between PDT and ferroptosis remains elusive. 

We previously found that multiple antibiotics combined with ALA_PDT can successfully cure patients with skin *M. abscessus* infections, whose ulcer surface gradually healed without recurrence [34]. This might be due to the bactericidal effect of ALA_PDT against *M. abscessus*. To explore whether ALA_PDT can kill *M. abscessus* and the underlying mechanism of action, an in vitro bactericidal model was established. We found that ALA_PDT could kill *M. abscessus* by promoting ROS-mediated bacterial ferroptosis-like death. The study provided new insights into the mechanism of action of ALA_PDT efficacy against *M. abscessus* and opened a new avenue to treat the antibiotics-recalcitrant *M. abscessus* infection.

## 2. Materials and Methods

### 2.1. Strains, Plasmids and Primers

The *M. abscessus* ATCC19977 strain was purchased from BeNa Culture Collection (Jiangshu, China). The *Escherichia coli* DH5α strain is used for gene cloning, grown in Luria-Bertani (LB) agar at 37 °C and contains appropriate antibiotics. *M. abscessus* was grown in 7H9 liquid medium or on 7H9 agar supplemented with 0.5% (*v/v*) glycerol and 0.05% (*v/v*) Tween 80. All strains were stored at −80 °C with 10% sterilized glycerol. The pALACE plasmid was kind gift of Professor Yossef Av-Gay at the University of British Columbia. The pALACE plasmid is an *E. coli-Mycobacterium* shuttle plasmid with a histidine tag. It is hygromycin resistant and is usually expressed under the induction of acetamide. The *M. abscessus* ATCC19977 genomic DNA was extracted, and the primers were synthesized by Beijing Genomics institution (Shenzhen, China). The list of strains and plasmids used in Table 1. Primers used in Table 2. 

### 2.2. The Effect of ALA_PDT on Bacteria Growth

The *M. abscesses* ATCC19977 strain was cultured in liquid 7H9 medium supplemented with 0.05% (*v/v*) Tween 80 and harvested when OD_600_ is 0.8, then adjusted OD_600_ to 0.1–0.2, and added the photosensitizer ALA (0–100 µg/mL). It was incubate at 37 °C for 12 h, and processed red light of 585–635 nm with different energy (0–160 J/cm^2^). *M. abscessus* was plated on 7H9 solid medium after gradient dilution, and the results were observed 4 days later. The *M. abscessus* ATCC19977 strain was cultured in liquid 7H9 medium supplemented with 0.05% (*v/v*) Tween 80 to an OD_600_ of about 0.8 and harvested. Then, the OD_600_ was adjusted to 0.1–0.2, and the photosensitizer ALA (100 µg/mL) was added at 37 °C and incubated for 12 h. The ROS scavenger N-Acetyl-L-cysteine, at a final concentration of 10mM, or the ferroptosis inhibitor Ferrostatin-1, at a final concentration of 20 µM, was added and incubated for 2 h at 37 °C, and then processed red light of 585–635 nm with different energy (80, 160 J/cm^2^). *M. abscessus* was diluted and inoculated on 7H9 solid medium, cultured in a constant temperature incubator at 37 °C, and the colonies were observed 4 days later.

### 2.3. ROS Measurement

Ctrl, PDT1, PDT2, PDT1 + NAC, PDT2 + NAC, PDT1 + Fer-1, PDT2 + Fer-1 (Ctrl: 0 µg/mL ALA + 0 J/cm^2^ light, PDT1: 100 µg/mL ALA + 80 J/cm^2^ light, PDT2: 100 µg/mL ALA + 160 J/cm^2^ light, PDT1 + NAC: 100 µg/Ml ALA + 80 J/cm^2^ light + 10 mM NAC, PDT2 + NAC: 100 µg/mL ALA + 160 J/cm^2^ light + 10 mM NAC, PDT1 + Fer-1: 100 µg/mL ALA + 80 J/cm^2^ light + 20 µM Ferrostatin-1, PDT2 + Fer-1: 100 µg/mL ALA + 160 J/cm^2^ light + 20 µM Ferrostatin-1) were centrifuged at 6800× *g* 10 min, the pellet was washed with 1×PBS 3 times, the OD_600_ was adjusted to about 0.4 and the ROS level was measured with a ROS Assay kit (Beyotime, Shanghai, China).

### 2.4. DNA Damage Measurement

Ctrl, PDT1 and PDT2 bacteria were collected by centrifugation, the pellet was washed 3 times in pre-cooled 1×PBS, adjusted OD_600_ = 0.8, took 500 μL of bacteria and added 500 μL of pre-cooled 4% paraformaldehyde. The bacteria were fixed for 30 min, the bacteria were collected, the pellet was washed 3 times with 1×PBS, bacteria were resuspended in 500 μL pre-cooled permeabilization solution, incubated at room temperature for 5 min and harvested by centrifugation. The pellet was washed with 1×PBS 3 times, and then measured according to the instruction of the One Step TUNEL Apoptosis Assay kit (Beyotime, Shanghai, China). 

### 2.5. Lipid Peroxidation Assay

The same amount of Ctrl, PDT1 and PDT2 bacteria was harvested, sonicated and centrifuged for 10 min at 6800× *g* and 4 °C. The supernatant was collected and placed on ice. Lipid peroxidation MDA Assay kit (Beyotime, Shanghai, China) was used for lipid peroxidation measurement.

### 2.6. Determination of Fe^2+^ Content in Bacteria

The same amount of Ctrl, PDT1 and PDT2 bacteria was harvested by centrifugation at 6800× *g* at 4 °C centrifugation for 10 min and sonicated. The resulting supernatant was collected and placed on ice. Quantichrom^TM^ iron detection kit (BioAssay Systems, Hayward, CA, USA) was used; the supernatant was incubated at room temperature for 40 min and a microplate reader (Molecular Device, San Jose, CA, USA) was used to read the optical density (peak absorbance at 590 nm) at wavelengths of 510–630 nm.

### 2.7. Determination of ATP Content in Bacteria

The Ctrl, PDT1 and PDT2 bacteria ATP content were measured with the ATP Assay kit (Beyotime, Shanghai, China) and the multifunctional microplate reader (Tecan, Männedorf, Switzerland).

### 2.8. Bacteria Intracellular NADH and NAD^+^ Determination

The bacteria content of NADH or NAD^+^ is detected by the Coenzyme I NAD (H) content detection kit (Solarbio, Beijing, China), and OD readings were performed with the microplate reader (Molecular Device, San Jose, CA, USA) at 570 nm.

### 2.9. RNA-seq

The Ctrl, PDT1 and PDT2 bacteria were harvested for RNA preparation. As mentioned before [35], RNA-seq is measured by Zhongke New Life (Shanghai, China). The original image data files obtained by high-throughput sequencing are converted into original sequenced reads (Sequenced Reads) by CASAVA base calling (Base Calling) analysis. The transcriptome sequencing (RNA-seq) data are aligned with the genome of the *M. abscess* ATCC 19977 in NCBI, and the gene expression level is estimated by the number of reads. To make it comparable between different genes or samples, the number of reads is converted to a counts value for normalization of gene expression. Gene expression data (accession number GSE193092) has been submitted to GEO (GENE EXPRESSION OMNIBUS).

### 2.10. qRT-PCR

For Ctrl, PDT1, and PDT2 strains, mRNA was collected and transcribed into cDNA. The following thermal cycling parameters were utilized for the PCR reaction (Bio-Rad IQ5): 95 °C for 5 min and 40 cycles, 95 °C for 30 s, 58 °C for 30 s and 72 °C for 30 s. Melting curve analysis was used to assess amplification specificity. The gene expression level was normalized to the sigA gene transcription level. The average relative expression level and standard deviation were determined from three independent experiments.

### 2.11. Construction of MAB_Vec and MAB_4773 Recombinant M. abscessus

*M. abscessus* ATCC 19977 genomic DNA was used as a template to amplify the MAB_4773 gene. The PCR product and the vector plasmid pALACE were digested to produce recombinant MAB_4773. All plasmids were electroporated into *M. abscessus*. After 15 h of in vitro growth in 7H9 liquid medium, the electroporated *M. abscessus* strain was inoculated on 7H9 agar containing hygromycin and cultured in a constant temperature incubator at 37 °C for 4–5 days.

### 2.12. Drug Resistance Analysis

Ctrl, PDT1, and PDT2 bacteria were collected and washed with 1×PBS buffer three times. The pellet was re-suspended in 7H9 medium, adjusted to OD_600_ of 0.4, followed by an addition of Nor: Norfloxacin 80, 160, 240 μg/mL, Cip: Ciprofloxacin 80, 160, 240 μg/mL, 10, 20, 30 μg/mL Cla: clarithromycin, 20, 40, 60 μg/mL and Min: Minocycline for 24 h pre-treatment. Bacterial suspensions were gradually diluted and treated bacteria were spread on 7H9 agar and incubated at 37 °C for 4 days.

### 2.13. Bacterial Membrane Integrity Test

To confirm the effect of ALA_PDT on the integrity of bacterial cell membranes, LIVE/DEAD^®^ BacLight^TM^ Bacterial Viability Kit L13152 (Thermo Fisher Scientific, Waltham, MA, USA) was used. SYTO 9 stain generally marks all the bacteria in the population, including those with intact or damaged cell membranes. Bacteria with intact cell membranes are more likely to stain fluorescent green. The excitation/emission maximum of these dyes is about 480/500 nm for SYTO 9 stain.

### 2.14. Nile Red Experiment

Ctrl, PDT1, and PDT2 bacteria were harvested and washed three times with PBST buffer (0.05% Tween80 added to 1×PBS). The pellet was resuspended in 7H9 medium, adjusted to an OD_600_ of 0.8, and 200 µL of bacterial suspension was added to a clean 96-well plate. Nile Red was added to corresponding wells to make sure their final concentrations were 2 µM. A multifunctional microplate reader (Tecan, Männedorf, Switzerland). was used to detect the fluorescence intensity every five min by setting the excitation spectrum to 544 nm and the emission spectrum to 590 nm.

### 2.15. Statistical Analysis

The experiment was carried out in triplicate. Prism 6 and Student’s *t*-test were used to analyze the differences between groups. *** *p* < 0.001, ** *p* < 0.01, * *p* < 0.05; n.s. is not significant; means ± standard deviation from at least three biological replicates.

## 3. Results

### 3.1. ALA_PDT Can Kill M. abscessus In Vitro

**Figure 1:**
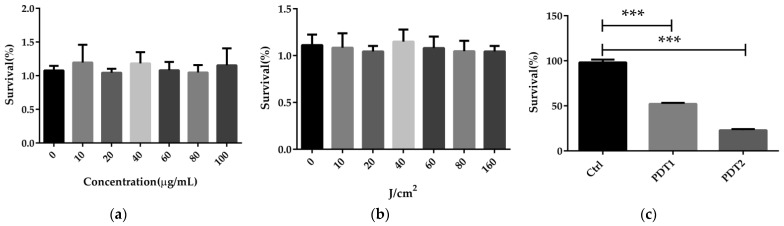
ALA_PDT can kill *M. abscessus* in vitro. (**a**) The effects of different concentrations of ALA on the growth of *M. abscesses*. (**b**) The effects of red light in different intensity on the growth of *M. abscessus*. (**c**) The effects of different concentrations of ALA_PDT on the growth of *M. abscessus*. (*** *p* < 0.001 and means ± standard deviation from at least three biological replicates).

ALA_PDT was previously reported to be able to inactivate a variety of bacteria in vitro, including mycobacteria [36]. To conclude whether ALA_PDT can kill *M. abscesses*, we first performed the sterilization experiment on *M. abscesses* in vitro. It was found that neither the photosensitizer ALA nor red light could kill *M. abscesses* (Figure 1a,b). A combination of photosensitizer and red light, PDT1 (100 µg/mL ALA + 80 J/cm^2^ red light) or PDT2 (100 µg/mL ALA + 160 J/cm^2^ red light) light eliminated *M. abscessus* in vitro in a dose-dependent manner (Figure 1c). The results demonstrated that ALA_PDT could kill *M. abscessus* in vitro.

### 3.2. ALA_PDT Promoted Ferroptosis-Like Death of M. abscessus

**Figure 2:**
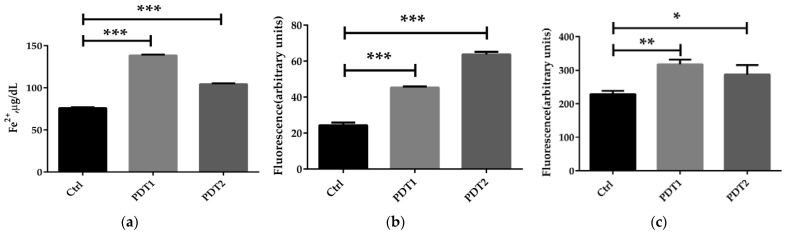

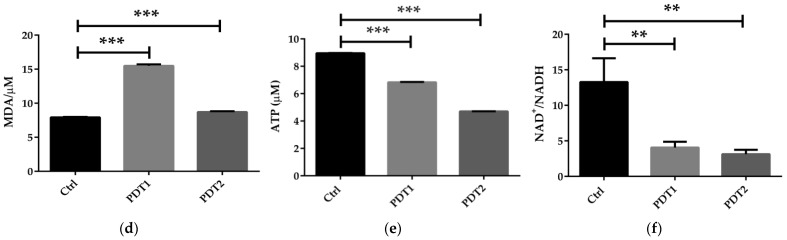
ALA_PDT promoted ferroptosis-like death of *M. abscessus*. (**a**) Determination of the total amount of Fe^2+^ in *M. abscessus* after ALA_PDT. (**b**) ROS level. (**c**) The degree of DNA damage. (**d**) Lipid peroxidation. (**e**) ATP content. (**f**) NAD^+^/NADH ratio. (*** *p* < 0.001, ** *p* < 0.01, * *p* < 0.05 and means ± standard deviation from at least three biological replicates).

To further to explore the mechanism underlying the effect of ALA_PDT on *M. abscessus*, we compared the related parameters in the Ctrl, PDT1 and PDT2, such as Fe^2+^ content, ROS level, DNA damage, lipid peroxidation, ATP amount and NAD^+^/NADH ratio. The results demonstrated that PDT1 and PDT2 significantly increased the Fe^2+^ content (Figure 2a), ROS level (Figure 2b), DNA damage (Figure 2c) and lipid peroxidation (Figure 2d) in the bacteria than the Ctrl. PDT lowered the ATP content (Figure 2e) and the NAD^+^/NADH ratio compared to control bacteria (Figure 2f). The data indicated that ALA_PDT might promote a ferroptosis-like death of *M. abscessus* by modulate the ferroptosis-related molecules.

### 3.3. ALA_PDT Promoted the Ferroptosis-Like Death of M. abscessus by Inducing the Production of ROS in Bacteria

**Figure 3:**
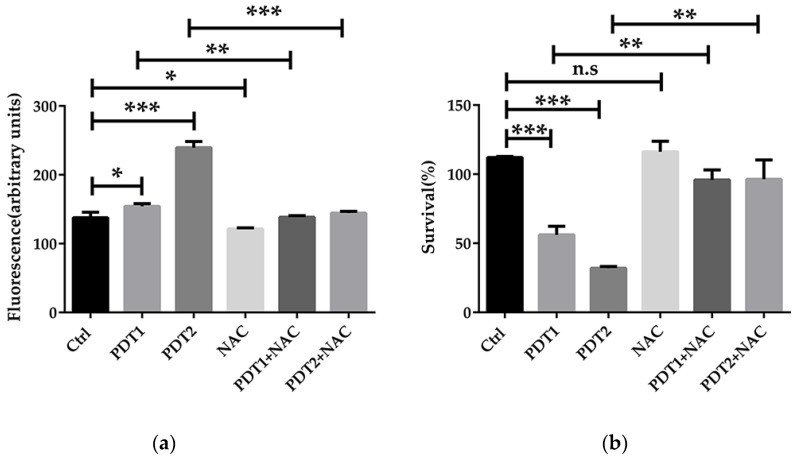

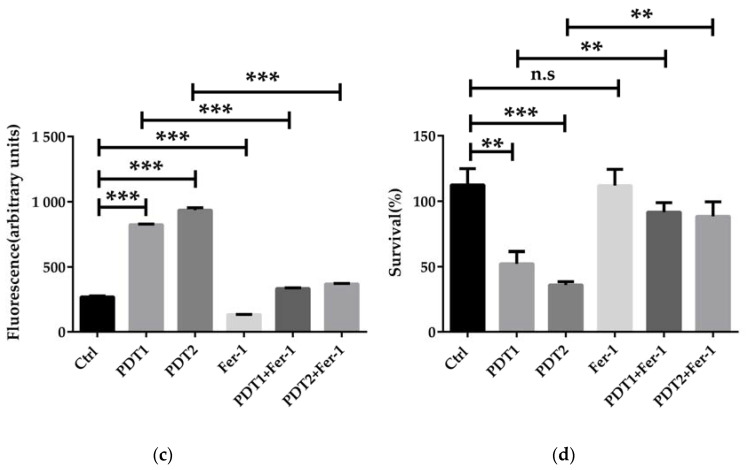
ALA_PDT inhibited the growth of *M. abscessus* by promoting ROS production. (**a**) Intracellular ROS content of ALA_PDT and ROS scavenger NAC in *M. abscessus*. (**b**) Effects of ALA_PDT and ROS scavenger NAC on the growth of *M. abscessus*. (**c**) Intracellular ROS content of ALA_PDT and ferroptosis inhibitor Fer-1 in *M. abscessus*. (**d**) Effects of ALA_PDT and ferroptosis inhibitor Fer-1 on the growth of *M. abscessus*. (*** *p* < 0.001, ** *p* < 0.01, * *p* < 0.05, n.s. is not significant and means ± standard deviation from at least three biological replicates).

To confirm the role of ferroptosis-like death in ALA_PDT effect on *M. abscessus,* ROS scavenger NAC and ferroptosis inhibitor Fer-1 were added before ALA_PDT. The antioxidant effect of NAC is mainly to quench ROS. Anti-ferroptotic activity of fer-1 is actually due to the scavenging of initiating alkoxyl radicals produced by Fe^2+^ from lipid hydroperoxides [37]. The results demonstrated that NAC decreased the production of ROS and rescued *M. abscessus* from death by ALA_PDT (Figure 3a,b), indicating that ALA_PDT played a role in the death of *M. abscessus* through the production of ROS. Fer-1 treatment can also reduce the production of ROS and bacteria killing by ALA_PDT (Figure 3c,d). The results demonstrated that ALA_PDT promoted *M. abscessus* ferroptosis-like death, not only via increasing the production of ROS, but also related to the change of the Fe^2+^ concentration in the bacteria.

### 3.4. ALA_PDT Up-Regulated the Transcriptional Level of Heme Oxygenase MAB_4773

**Figure 4:**
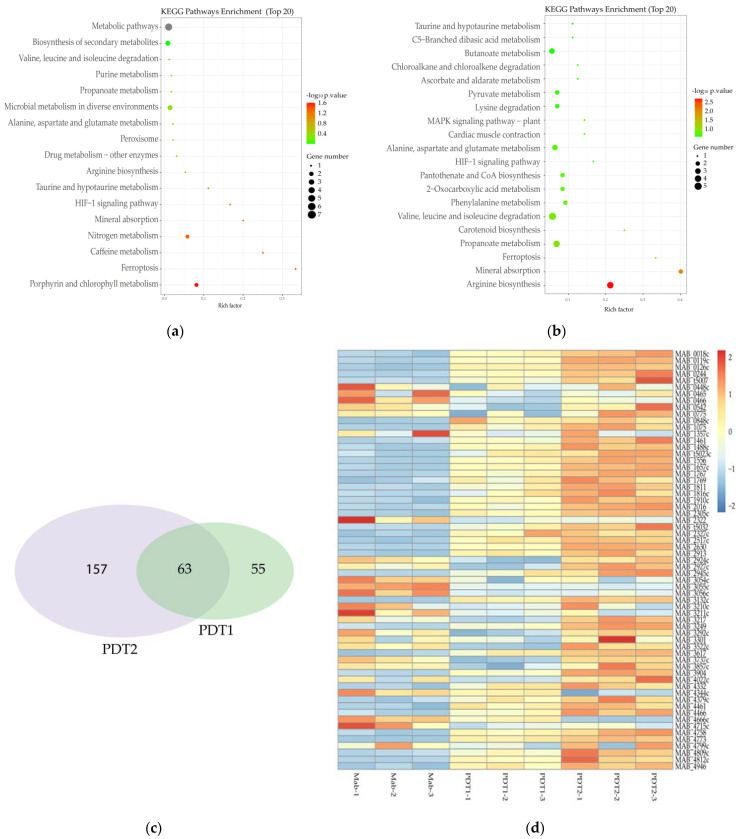
Transcriptome change of *M. abscessus* upon ALA_PDT. (**a**) Transcriptome KEGG pathway enrichment analysis of Ctrl and PDT1. (**b**) Transcriptome KEGG pathway enrichment analysis of Ctrl and PDT2. (**c**) Venn diagram of PDT1 and PDT2; the number of genes that change in the transcriptome of *M. abscessus*. (**d**) Heat map of related genes that change together in the transcriptome of Ctrl, PDT1 and PDT2.

To further define the specific mechanism of action underlying the effect of ALA_PDT on *M. abscessus*, transcriptome of *M. abscessus* after PDT1 or PDT2 was determined by RNA-sequencing. The results demonstrated that PDT1 significantly affected the transcription of 118 *M. abscessus* genes, of which 68 genes were up-regulated and 50 genes were down-regulated. The KEGG pathway enrichment analysis demonstrated that the ALA_PDT-regulated genes are involved in multiple metabolic processes (Figure 4a). Some genes are: nitrogen metabolism-related genes, MAB_4344c and MAB_3522c; porphyrin metabolism-related genes, MAB_2986c and MAB_4773; arginine biosynthesis; alanine, aspartic acid and glutamate metabolism-related genes, MAB_4344c, valine and leucine; and isoleucine degradation-related genes, MAB_4539c. PDT2 significantly changed the transcription of 220 genes, of which 113 genes were up-regulated and 107 genes were down-regulated. PDT2 changed the genes involved in the biosynthesis and metabolism of amino acids, such as the degradation of valine, leucine, isoleucine and lysine, the biosynthesis of arginine and the metabolism of alanine, aspartate and glutamate (Figure 4b). There are 63 genes demonstrating the same change trend upon PDT1 or PDT2 treatment. The detailed results are shown in Figure 4c. The amino acid metabolism and ferroptosis pathways are significantly enriched in PDT1 or PDT2 differentially regulated genes, such as heme oxygenase, MAB_4773. The results indicate that ALA_PDT may alter the metabolism of amino acids and ferroptosis. Heme oxygenase is involved in the ferroptosis of cells [38]. The transcription of heme oxygenase-encoding gene MAB_4773 in *M. abscessus* was significantly up-regulated after ALA_PDT (Figure 4d), which might be important in the promotion of ferroptosis-like death of *M. abscessus* by ALA_PDT. 

### 3.5. ALA_PDT Promoted Bacterial Ferroptosis-Like Death by MAB_4773

**Figure 5:**
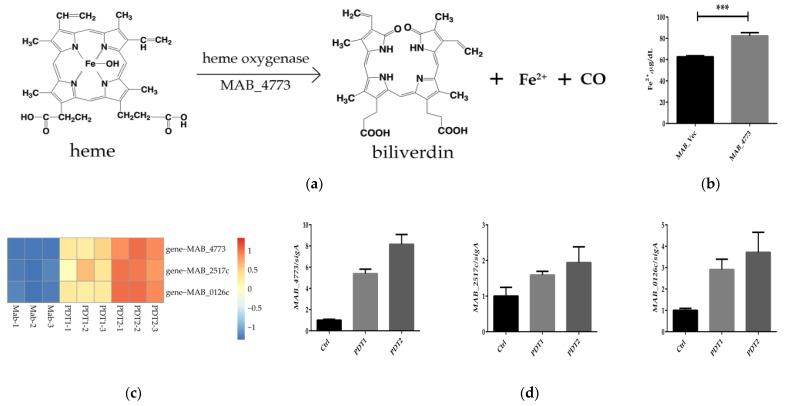
MAB_4773 affects iron metabolism in *M. abscessus*. (**a**) Heme oxygenase decomposes heme. (The figure only presents the Fe^2+^ generation). (**b**) The Fe^2+^ level of MAB_Vec and MAB_4773. (**c**) Heat map of transcription levels of iron metabolism-related genes. (**d**) qRT-PCR results of transcription levels of iron metabolism-related genes. (*** *p* < 0.001 and means ± standard deviation from at least three biological replicates).

Heme oxygenase (HO) is the rate-limiting enzyme of heme catabolism, which can decompose heme to produce biliverdin, CO and Fe^2+^ (Figure 5a). HO-mediated iron release is the major intracellular source of labile iron; HO is involved in the ferroptosis of cells [38]. In order to prove the important role of MAB_4773 in the ferroptosis-like death caused by ALA_PDT, we overexpressed MAB_4773 in vitro. The results demonstrated that MAB_4773 overexpression recombinant *M. abscesses* did accumulate higher Fe^2+^ (Figure 5b), consistent with ALA_PDT causing ferroptosis-like death by up-regulating the transcription of MAB_4773. Heme oxygenase MAB_4773, bacterial ferritin MAB_0126c, low-affinity iron permease MAB_2517c and other genes related to bacterial iron metabolism were also up-regulated in the transcriptome (Figure 5c), consistent with in vitro qRT-PCR results (Figure 5d). The results demonstrated that ALA_PDT can alter *M. abscessus* intracellular iron metabolism by up-regulating the transcription of MAB_4773, thereby promoting bacteria ferroptosis-like death.

### 3.6. ALA_PDT Disrupts the Integrity of the Cell Membrane and Potentiates the Sterilization of Antibiotics

**Figure 6:**
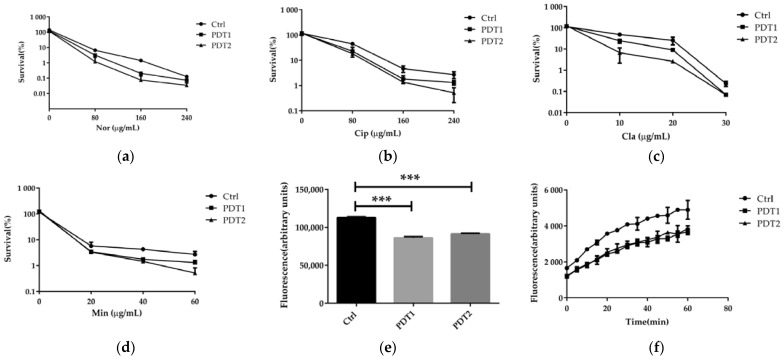
ALA_PDT potentiated antibiotics effect against *M. abscessus*. (**a**–**d**) ALA_PDT increases antibiotic sterilization against *M. abscessus*. (**e**) The cell membrane integrity of *M. abscessus*. (**f**) The Nile Red fluorescent dye accumulation in *M. abscessus*. (Nor: Norfloxacin, Cip: Ciprofloxacin, Cla: clarithromycin and Min: Minocycline. *** *p* < 0.001 and means ± standard deviation from at least three biological replicates).

We next explored whether a combination of ALA_PDT and antibiotics can reduce NTM infections, or whether ALA_PDT can boost the efficacy of antibiotics against *M. abscessus*. We compared the effect of antibiotics on bacteria with or without ALA_PDT treatment in vitro. The results demonstrated that ALA_PDT potentiated the killing effect of antibiotics, such as norfloxacin, ciprofloxacin, clarithromycin, and minocycline, against *M. abscessus* (Figure 6a–d). Ferroptosis was reported to disrupt cell membrane integrity [39]. We speculated that the cell membrane integrity corruption might underlie the potentiation effect of antibiotics. To confirm this, SYTO 9 dye was used to detect the integrity of the cell membrane. We found that ALA_PDT significantly reduced the integrity of the cell membrane (Figure 6e). Subsequent permeability change might underlie the potentiation of antibiotics. The dye Nile Red test demonstrated that ALA_PDT did increase the permeability of cell membranes (Figure 6f). In summary, ALA_PDT reduced the integrity and increased the permeability of the cell membrane, resulting in a boost of the antibiotic effect on *M. abscessus*.

## 4. Discussion

We demonstrated that ALA_PDT can kill *M. abscessus* and promote the sterilization effect of antibiotics by promoting ferroptosis-like death of *M. abscessus*. This provided a new mechanistic understanding as to the efficacy of ALA_PDT against *M. abscessus*, and rationale for ALA_PDT in clinical treatment of *M. abscessus* infection.

We firstly demonstrated that ALA_PDT could kill *M. abscessus* through bactericidal experiments in vitro, followed by demonstrating that ALA_PDT can promote *M. abscessus* ferroptosis-like death. By using ROS quenchers and ferroptosis inhibitors, we found that ALA_PDT could promote *M. abscessus* ferroptosis-like death by increasing the production of ROS. RNA-seq transcriptome demonstrated that ALA_PDT up-regulated the transcription of genes involved in iron metabolism, including heme oxygenase MAB_4773. MAB_4773 overexpression recombinant increases the amount of Fe^2+^, further confirming that MAB_4773 can promote *M. abscessus* ferroptosis-like death by affecting bacterial iron metabolism and engaging in ALA_PDT. ALA_PDT can potentiate the efficacy of multiple antibiotics, largely via changing the *M. abscessus* cell membrane permeability.

The bactericidal effect of ALA_PDT was previously documented [14,40], but the underlying mechanism of action remains elusive. Mycobacteria can reprogram its metabolism to adapt with the dynamic host environment [41], such as oxidative stress. We found that ALA_PDT can promote bacterial ferroptosis-like death by producing ROS, and affect the bacterial iron metabolism by up-regulating the transcription of MAB_4773 via a hitherto unknown mechanism of action. This study supplies new insights into the bactericidal mechanism of ALA_PDT. For the first time, we linked the ferroptosis-like death with the bactericidal mechanism of ALA_PDT. ALA_PDT killed bacteria by ROS generated in or near the bacteria. However, we firstly propose that ALA_PDT causes bacterial ferroptosis-like death by promoting the production of ROS. Heme oxygenase can directly alter the bacteria’s ability to resist oxidative damage [42,43] and is related to ferroptosis [44]. Bacterial heme oxygenase function was largely assigned to the degradation of heme [45,46]. *M. abscessus* contains a heme oxygenase, MAB_4773. We found that ALA_PDT up-regulated heme oxygenase MAB_4773 and is directly related to the production of ROS. Whether ALA_PDT can change the *M. abscessus* heme quantity, and how this functions in the bactericidal effect, remains to be determined. We found that ALA_PDT may alter the iron metabolism of bacteria by up-regulating the transcription of MAB_4773, thereby promoting *M. abscessus* ferroptosis-like death. However, how MAB_4773 specifically affects iron metabolism and ferroptosis-like death in *M. abscessus* warrants further study. The presence of heme oxygenase in other bacteria [47] implicates that ALA_PDT might have wider application for bacterial infection.

*M. abscessus* infection is an emerging public health concern with its intrinsic drug resistance, which necessitates the prolonged administration of multiple antibiotics [48], which greatly limited the choice of treatments [49]. We found that ALA_PDT reduced the drug resistance of *M. abscessus* by increasing the permeability of the cell membrane. This may underlie the broad-spectrum efficacy of ALA_PDT in combination with antibiotics. ALA_PDT might be a potentiating factor of antibiotics against other bacterial infections. Though very few antibiotics, such as norfloxacin, ciprofloxacin, clarithromycin and minocycline, were included in this study, other antibiotics in the clinical guidance can be further tested. 

*M. abscessus* causes a large number of infections worldwide, often with underestimated disease burden. Multidrug resistant (MDR) and extensive drug resistant (XDR) pathogens have become a serious threat to public health [50]. The rapid increase in antibiotic resistant bacteria has neutralized the efficacy of many antibiotics. Many NTM infections are characterized by relapse and drug resistance [51]. ALA_PDT is safe and easy to implement, and effective against bacteria [52], fungi [53], viruses [54,55] and protozoa [56,57]. Compared with conventional antibiotics, ALA_PDT acts rapidly and can even sterilize drug resistant strains [58,59]. This broad-spectrum effect might have an important role in the treatment of emerging infectious diseases. ALA_PDT for *M. abscessus* treatment is not routine in clinical practice. Our study provides new options and rationale for the treatment of *M. abscessus* infections with ALA_PDT. The study of the mechanism of ALA_PDT killing *M. abscessus* can inspire the use of ALA_PDT against other mycobacteria infection, and even more pathogens. Further animal experiments and clinical experiments are needed to support the wider application of ALA_PDT.

We found that the same dose of ALA_PDT can damage cells; whether this damage will affect the application of ALA_PDT requires further experiments in eukaryotic cells and mice. The detailed pathway and genes involved shall be further explored to define the molecular basis, and to establish the utility of ALA_PDT in clinical practice.

## 5. Conclusions

In conclusion, although our study is limited to in vitro experiments, this well demonstrated that ALA_PDT could kill *M. abscessus* and promoted the sterilization effect of antibiotics by promoting ferroptosis-like death. Meanwhile, we demonstrated that ALA_PDT could promote ferroptosis-like death in *M. abscessus* by increasing ROS production, which may alter bacterial iron metabolism by up-regulating the transcription of MAB_4773. Disruption of the cell membrane integrity can be elicited by ALA_PDT, accompanied by bacterial ferroptosis-like death. This might have broader applications for the ALA_PDT potentiating effect on antibiotics.

## Figures and Tables

**Table 1:** The list of strains and plasmids used in the study.

Strains	Description of Strains	Source
WT	Wild type *M. abscessus* ATCC 19977 strain	This study
MAB_Vec	*M.abscessus* with transformed with vector pALACE	This study
MAB_4773	*M.abscessus* with transformed with vector pALACE_MAB_4773	This study
*E. coli* DH5a	Strain used in vector proliferation	Invitrogen
Plasmids	Description of Plasmids	
pALACE	A replicative plasmid used for gene expression in *M. abscessus* and conferring hygromycin (hyg) resistance	

**Table 2:** Primers used in the study.

Primer	Description	Sequence of Primers (5′-3′)	Gene ID
MAB_4773 F	Construct recombinant MAB_4773 strain	GGAATTCCATATGATGAACGTCAGCTCTTCCACGGTTC	5967230
MAB_4773 R		CCATCGATCAGCGCCGGTAGGCGGTCAGATTG	
MAB_4773 RT-F	RT-PCR	AGTCGCCGTTCATCTCCGAACTGCTTGC	
MAB_4773 RT-R		CGTCTTCGATGGCGGTGTAGATCATCTGTAA	
MAB_0126c RT-F		CACGAGTTCACGGCATCGCAGCAATACA	5962669
MAB_0126c RT-R		TTTTTGGGGTGTCGGTCGCGGTCAT	
MAB_2517c RT-F		TACCTGGCAGTTGGTCATCAACACCTTG	5965027
MAB_2517c RT-R		GTTGAGCTTCTGCTGGACCGCGTCATCG	

## Data Availability

The data presented in this study are available in this manuscript.

## References

[1] Johansen M.D., Herrmann J.L., Kremer L. (2020). Non-tuberculous mycobacteria and the rise of Mycobacterium abscessus. Nat. Rev. Microbiol..

[2] Falkinham J.O. (2015). Environmental sources of nontuberculous mycobacteria. Clin. Chest Med..

[3] Howard S.T., Byrd T.F. (2000). The rapidly growing mycobacteria: Saprophytes and parasites. Microbes Infect..

[4] Rachid N., Emmanuelle C., Jean M.R., Alan M., Brigitte G. (2012). Mycobacterium abscessus: A new antibiotic nightmare. J. Antimicrob. Chemoth..

[5] Johansen M.D., Alcaraz M., Dedrick R.M., Roquet-Banères F., Hamela C., Hatfull G.F., Kremer L. (2021). Mycobacteriophage–antibiotic therapy promotes enhanced clearance of drug-resistant Mycobacterium abscessus. Dis. Models Mech..

[6] Dedrick R.M., Guerrero-Bustamante C.A., Garlena R.A., Russell D.A., Spencer H. (2019). Engineered bacteriophages for treatment of a patient with a disseminated drug-resistant Mycobacterium abscessus. Nat. Med..

[7] Umadevi S.S., Linh T.T. (2001). P-113D, an antimicrobial peptide active against Pseudomonas aeruginosa, retains activity in the presence of sputum from cystic fibrosis patients. Antimicrob. Agents Chem..

[8] Wainwright M. (1998). Photodynamic antimicrobial chemotherapy (PACT). J. Antimicrob. Chemoth..

[9] Jori G., Fabris C., Soncin M., Ferro S., Coppellotti O., Dei D., Fantetti L., Chiti G., Roncucci G. (2006). Photodynamic therapy in the treatment of microbial infections: Basic principles and perspective applications. Laser. Surg. Med..

[10] Shleeva M., Savitsky A., Kaprelyants A. (2021). Photoinactivation of mycobacteria to combat infection diseases: Current state and perspectives. Appl. Microbiol. Biotechnol..

[11] Banks J.G., Board R.G., Carter J., Dodge A.D. (1985). The cytotoxic and photodynamic inactivation of micro-organisms by Rose Bengal. J. Appl. Microbiol..

[12] Sung N., Back S., Jung J., Kim K.H., Kim J.K., Lee J.H., Ra Y., Yang H.C., Lim C., Cho S. (2013). Inactivation of multidrug resistant (MDR)-and extensively drug resistant (XDR)-Mycobacterium tuberculosis by photodynamic therapy. Photodiagn. Photodyn..

[13] Vallejo M., Moura N.M., Gomes A.T., Joaquinito A.S. (2021). The Role of Porphyrinoid Photosensitizers for Skin Wound Healing. Int. J. Mol. Sci..

[14] Harris F., Pierpoint L. (2012). Photodynamic therapy based on 5-aminolevulinic acid and its use as an antimicrobial Agent. Med. Res. Rev..

[15] Ji E.C., Chul-Ho O., Nackmoon S., Sanghoon J. (2015). The potential application of photodynamic therapy in drug-resistant tuberculosis. J. Photoch. Photobio. B.

[16] Gong N., Tan Y., Li M., Lu W., Lei X. (2016). ALA-PDT combined with antibiotics for the treatment of multiple skin abscesses caused by Mycobacterium fortuitum. Photodiagn. Photodyn. Ther..

[17] O’Riordan K., Akilov O.E., Chang S.K., Foley J.W., Hasan T. (2007). Real-time fluorescence monitoring of phenothiazinium photosensitizers and their anti-mycobacterial photodynamic activity against Mycobacterium bovis BCG in in vitro and in vivo models of localized infection. Photoch. Photobio. Sci..

[18] Elke F., Reza G. (2009). Highly efficient in vitro photodynamic inactivation of Mycobacterium smegmatis. J. Antimicrob. Chemoth..

[19] O’Riordan K., Sharlin D.S., Gross J., Chang S., Errabelli D., Akilov O.E., Kosaka S., Nau G.J., Hasan T. (2006). Photoinactivation of Mycobacteria In Vitro and in a New Murine Model of Localized Mycobacterium bovis BCG-Induced Granulomatous Infection. Antimicrob. Agents Chem..

[20] Sun K., Yang H., Huang X., Gong N., Qin Q., Lu W., Lei X. (2017). ALA-PDT combined with antibiotics for the treatment of atypical mycobacterial skin infections: Outcomes and safety. Photodiagn. Photodyn. Ther..

[21] Maisch T., Hackbarth S., Regensburger J. (2011). Photodynamic inactivation of multi-resistant bacteria (PIB)—A new approach to treat superficial infections in the 21st century. J. Deutsch. Dermatol. Ges..

[22] Plaetzer K., Krammer B., Berlanda J., Berr F., Kiesslich T. (2009). Photophysics and photochemistry of photodynamic therapy: Fundamental aspects. Laser. Med. Sci..

[23] Phoenix D., Harris F. (2006). Light activated compounds as antimicrobial agents-patently obvious?. Recent Pat. Antiinfect. Drug Discov..

[24] Davies M.J. (2004). Reactive species formed on proteins exposed to singlet oxygen. Photoch. Photobiol. Sci..

[25] Davies M.J. (2005). The oxidative environment and protein damage. BBA-Proteins Proteom..

[26] Girotti A.W. (2001). Photosensitized oxidation of membrane lipids: Reaction pathways, cytotoxic effects, and cytoprotective mechanisms. J. Photoch. Photobiol. B.

[27] Girotti A.W., Kriska T. (2004). Role of lipid hydroperoxides in photo-oxidative stress signaling. Antioxid. Redox Sign..

[28] Menezes S., Capella M., Caldas L.R. (1990). Photodynamic action of methylene blue: Repair and mutation in Escherichia coli. J. Photoch. Photobio. B.

[29] Imray F.P., Macphee D.G. (1973). The role of DNA polymerase I and the rec system in survival of bacteria and bacteriophages damaged by the photodynamic action of acridine orange. Mol. Gen. Genet..

[30] Shui S., Zhao Z., Wang H., Conrad M., Liu G. (2021). Non-enzymatic lipid peroxidation initiated by photodynamic therapy drives a distinct ferroptosis-like cell death pathway. Redox Biol..

[31] Dixon S.J., Lemberg K.M., Lamprecht M.R., Skouta R., Zaitsev E.M., Gleason C.E., Patel D.N., Bauer A.J., Cantley A.M., Yang W.S. (2012). Ferroptosis: An iron-dependent form of nonapoptotic cell death. Cell.

[32] Xie Y., Hou W., Song X., Yu Y., Huang J., Sun X., Kang R., Tang D. (2016). Ferroptosis: Process and function. Cell Death Differ..

[33] Shen X., Ma R., Huang Y., Chen L., Xu Z., Li D., Meng X., Fan K., Xi J., Yan X. (2020). Nano-decocted ferrous polysulfide coordinates ferroptosis-like death in bacteria for anti-infection therapy. Nano Today.

[34] Sun K., Li J., Li L., Li G., Wang L., Chen J., Wu X., Luo J., Liu H., Wang X. (2021). A new approach to the treatment of nontuberculous mycobacterium skin infections caused by iatrogenic manipulation: Photodynamic therapy combined with antibiotics: A pilot study. Photodiagn. Photodyn. Ther..

[35] Li Q., Zhou M., Fan X., Yan J., Li W., Xie J. (2016). Mycobacteriophage SWU1 gp39 can potentiate multiple antibiotics against Mycobacterium via altering the cell wall permeability. Sci. Rep..

[36] Shleeva M.O., Savitsky A.P., Nikitushkin V.D., Soloviev I.D., Kaprelyants A.S. (2020). Effect of Photodynamic Inactivation against Dormant Forms and Active Growing Cells of Mycobacterium smegmatis. Appl. Biochem. Microbiol..

[37] Miotto G., Rossetto M., DiPaolo M.L., Orian L., Venerando R., Roveri A., Vučković A.M., Travain V.B., Zaccarin M., Zennaro L. (2020). Insight into the mechanism of ferroptosis inhibition by ferrostatin-1. Redox Biol..

[38] NaveenKumar S.K., SharathBabu B.N., Hemshekhar M., Kemparaju K., Girish K.S., Mugesh G. (2018). The role of reactive oxygen species and ferroptosis in heme-mediated activation of human platelets. ACS Chem. Biol..

[39] Yan B., Ai Y., Sun Q., Ma Y., Cao Y., Wang J., Zhang Z., Wang X. (2021). Membrane damage during ferroptosis is caused by oxidation of phospholipids catalyzed by the oxidoreductases POR and CYB5R1. Mol. Cell.

[40] Barra F., Roscetto E., Soriano A.A., Vollaro A., Postiglione I., Pierantoni G.M., Palumbo G., Catania M.R. (2015). Photodynamic and antibiotic therapy in combination to fight biofilms and resistant surface bacterial infections. Int. J. Mol. Sci..

[41] Kumar A., Farhana A., Guidry L., Saini V., Hondalus M., Steyn A. (2011). Redox homeostasis in mycobacteria: The key to tuberculosis control?. Expert Rev. Mol. Med..

[42] Chen K., Gunter K., Maines M.D. (2000). Neurons overexpressing heme oxygenase-1 resist oxidative stress-mediated cell death. J. Neurochem..

[43] Wu J., Li S., Li C., Cui L., Ma J., Hui Y. (2021). The non-canonical effects of heme oxygenase-1, a classical fighter against oxidative stress. Redox Biol..

[44] Kwon M.Y., Park E., Lee S.J., Chung S.W. (2015). Heme oxygenase-1 accelerates erastin-induced ferroptotic cell death. Oncotarget.

[45] Ratliff M., Zhu W., Deshmukh R., Wilks A., Stojiljkovic I. (2001). Homologues of neisserial heme oxygenase in gram-negative bacteria: Degradation of heme by the product of the pigA gene of Pseudomonas aeruginosa. J. Bacteriol..

[46] Kikuchi G., Yoshida T., Noguchi M. (2005). Heme oxygenase and heme degradation. Biochem. Biophys. Res. Commun..

[47] Guo Y., Guo G., Mao X., Zhang W., Xiao J., Tong W., Liu T., Xiao B., Liu X., Feng Y. (2008). Functional identification of HugZ, a heme oxygenase from Helicobacter pylori. BMC Microbiol..

[48] Wang S.H., Pancholi P. (2014). Mycobacterial skin and soft tissue infection. Curr. Infect. Dis. Rep..

[49] Lopeman R.C., Harrison J., Desai M., Cox J.A. (2019). Mycobacterium abscessus: Environmental bacterium turned clinical nightmare. Microorganisms.

[50] Tanwar J., Das S., Fatima Z., Hameed S. (2014). Multidrug resistance: An emerging crisis. Interdiscip. Perspect. Infect. Dis..

[51] Jeon K., Kwon O.J., Lee N.Y., Kim B.J., Kook Y.H., Lee S.H., Park Y.K., Kim C.K., Koh W.J. (2009). Antibiotic treatment of Mycobacterium abscessus lung disease: A retrospective analysis of 65 patients. Am. J. Resp. Crit. Care Med..

[52] Tan Y., Cheng Q., Yang H., Li H., Gong N., Liu D., Wu J., Lei X. (2018). Effects of ALA-PDT on biofilm structure, virulence factor secretion, and QS in Pseudomonas aeruginosa. Photodiagn. Photodyn. Ther..

[53] Tegos G., Dai T., Fuchs B.B., Coleman J.J., Prates R.A., Astrakas C., StDenis T.G., Ribeiro M.S., Mylonakis E., Hamblin M.R. (2012). Concepts and principles of photodynamic therapy as an alternative antifungal discovery platform. Front. Microbiol..

[54] Costa L., Tomé J., Neves M., Tomé A., Cavaleiro J., Faustino M., Cunha N., Gomes N., Almeida A. (2011). Evaluation of resistance development and viability recovery by a non-enveloped virus after repeated cycles of aPDT. Antivir. Res..

[55] Banerjee I., Douaisi M.P., Mondal D., Kane R.S. (2012). Light-activated nanotube-porphyrin conjugates as effective antiviral agents. Nanotechnology.

[56] Akilov O.E., Kosaka S., O’Riordan K., Song X., Sherwood M., Flotte T.J., Foley J.W., Hasan T. (2006). The Role of Photosensitizer Molecular Charge and Structure on the Efficacy of Photodynamic Therapy against Leishmania Parasites. Chem. Biol..

[57] Wainwright M., Maisch T., Nonell S., Plaetzer K., Almeida A., Tegos G.P., Hamblin M.R. (2016). Photoantimicrobials-are we afraid of the light?. Lancet Infect. Dis..

[58] Huang J., Guo M., Jin S., Wu M., Yang C., Zhang G., Wang P., Ji J., Zeng Q., Wang X. (2019). Antibacterial photodynamic therapy mediated by 5-aminolevulinic acid on methicillin-resistant Staphylococcus aureus. Photodiagn. Photodyn. Ther..

[59] Yang Z., Feng Y., Pang Z., Li D., Wang S., Chen H., Jiang M., Yan H., Li T., Fu H. (2021). 5-aminolevulinic acid-photodynamic therapy ameliorates cutaneous granuloma by killing drug-resistant Mycobacterium marinum. bioRxiv.

